# Chagas Disease Screening in Maternal Donors of Publicly Banked Umbilical Cord Blood, United States

**DOI:** 10.3201/eid2208.151622

**Published:** 2016-08

**Authors:** James M. Edwards, Jennifer B. Gilner, Jose Hernandez, Joanne Kurtzberg, R. Phillips Heine

**Affiliations:** Duke University Medical Center, Durham, North Carolina, USA

**Keywords:** Chagas disease, vertical infection transmission, umbilical cord blood, parasitic diseases, vector-borne infections, Trypanosoma cruzi, parasites

## Abstract

To assess patterns of Chagas disease, we reviewed results of screening umbilical cord blood from a US public cord blood bank during 2007–2014. Nineteen maternal donors tested positive for *Trypanosoma cruzi* parasites (0.04%). Because perinatal transmission of Chagas disease is associated with substantial illness, targeted prenatal programs should screen for this disease.

Chagas disease, a parasitic disease caused by *Trypanosoma cruzi*, is increasingly seen in non–disease-endemic areas, secondary to population movements (*1*). Vertical transmission of this parasite to a developing fetus occurs at a rate of ≈4.7% and can cause substantial perinatal illness and death (*2*). Adverse pregnancy-related outcomes include increased rates of preterm delivery, restricted fetal growth, low birthweight, premature rupture of membranes, and polyhydramnios (*3*). Mortality rates among congenitally infected infants approach 5%, mostly secondary to myocarditis and meningoencephalitis. Long-term maternal and child conditions include dilated cardiomyopathy and gastrointestinal disorders, with mortality rates as high as 13% (*4*). Fortunately, infants within the first year of life tolerate treatment well, and the infection is usually cured (*3*).

Because screening for and treating this infection has potential benefits, the World Health Assembly adopted a resolution recommending screening for Chagas disease among pregnant women in non–disease-endemic areas who were born in disease-endemic areas, who lived extensively in disease-endemic areas, or who were born to mothers who lived in disease-endemic areas (*5*). The US Food and Drug Administration instituted guidelines for screening in 2007 (*6*). This screening also applied to mothers donating their newborn infants’ cord blood to public cord blood banks. Therefore, we reviewed identified cases of Chagas disease in maternal donors to a public umbilical cord blood bank to estimate disease prevalence and population characteristics in a non–disease-endemic area of the United States.

## The Study

We performed a retrospective cohort study of the seroprevalence of *Trypanosoma cruzi* parasites in all cord blood samples donated to the Carolinas Cord Blood Bank (CCBB) during July 1, 2007–December 31, 2014. The CCBB is a public cord blood bank (licensed by the US Food and Drug Administration) that collects donations from multiple sites across the state of North Carolina as well as from Boston, Massachusetts, and Atlanta, Georgia. A kit donation program also enables donations to be made from any US state. Initial donor screening selects patients with singleton, nonanomalous pregnancies without known preexisting infection. After CCBB received general written informed consent for cord blood donation at the time of delivery, we assessed blood samples from mothers whose cord blood donations met specifications of initial donor screening, volume, and cell count. Donor demographic information, including maternal age, race, ethnic background, state of collection, and date of collection, was recorded.

Maternal blood samples from cord blood donors were routinely screened for infectious agents at the American Red Cross National Donor Testing Laboratory (Charlotte, NC, USA). These agents were hepatitis B, hepatitis C, HIV-1 and -2, human T-lymphotrophic viruses I and II, *Treponema pallidum* (for syphilis, by rapid plasma regain test), cytomegalovirus, West Nile virus, *T. cruzi*, and any bacterial contamination. *T. cruzi* screening was performed by indirect hemagglutination assay. If results were positive, a confirmatory radioimmunoprecipitation assay (RIPA) was performed. Positive confirmatory testing triggered referral of mother and neonate for further evaluation and treatment. Because CCBB records are not linked to patient records, we were unable to obtain follow-up information regarding additional maternal and neonatal evaluation and treatment. After the study received exempt status from the Duke University Institutional Review Board (Pro00064159), we performed a retrospective cohort study and collected demographic data from mothers whose umbilical cord blood donations were positive for *T. cruzi*. Descriptive statistics were then performed and results were analyzed. All statistical analyses were performed with R version 3.2.2 (https://www.r-project.org/).

We screened samples from 58,817 maternal donors who donated cord blood during the 8.5-year period covered by the study. Twenty-five samples were positive by indirect hemaglutination assay (0.043%), and 19 were positive by confirmatory RIPA testing (0.032%). For 3 donors, confirmatory RIPA results were indeterminate, and results for 3 other donors were negative. One donor with a positive confirmatory test result had a positive rapid plasma regain result, and another was co-infected with hepatitis C virus. The remaining 17 donors from the cohort had no identified co-infections.

Of the donors with positive samples, 20 were from North Carolina, 1 was from Florida, 1 was from Massachusetts, and 3 were of unknown origin. When the total screened population was assessed, a substantial amount of missing data precluded the possibility of further analysis. We also attempted to assess maternal age but were unable to do so because of missing data.

The incidence of confirmed Chagas disease among mothers who donated their neonate’s cord blood varied over time, from an incidence of 0.3 cases/1,000 donors in 2007 to 1.6 cases/1,000 donors in 2014 (Figure). The ethnic distribution of donors with confirmed positive results was significantly different from that of the full population of screened donors (p = 0.002) (Table). The primary difference was that the number of Hispanic patients increased as the numbers of African American case-patients and Asian–Pacific Islander case-patients decreased.

**Figure:**
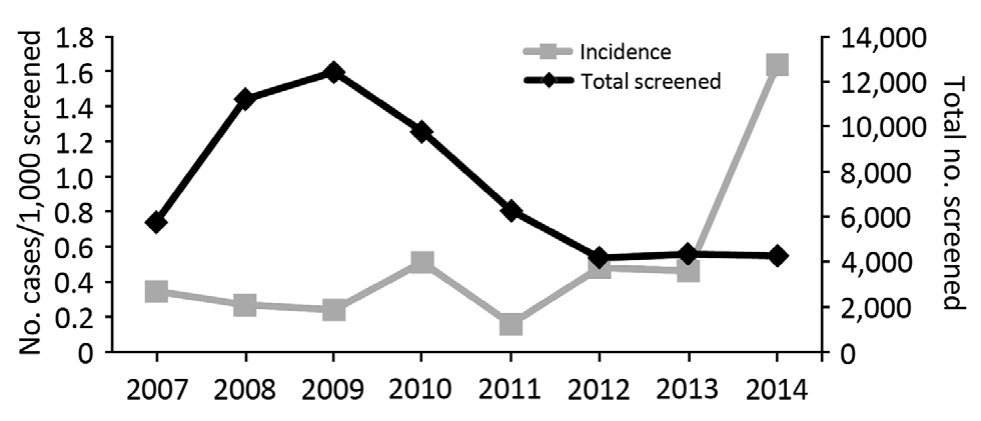
Chagas disease incidence in donated cord blood, United States, 2007–2014.

**Table:** Distribution by ethnicity of maternal cord blood donors with confirmed Chagas disease, United States, 2007–2014

Ethnicity	No. (%) patients with Chagas disease, n = 19	No. (%) screened donors
Caucasian	8 (32)	30,332 (52)
Hispanic	7 (28)	9,112 (16)
African American	2 (8)	10,277 (18)
Asian–Pacific Islander	0	1,833 (3)
Other	0	5,387 (10)
Unknown	2 (8)	854 (1)

## Conclusion

Chagas disease is an emerging infection in non–disease-endemic regions, such as the United States, secondary to emigration from disease-endemic areas, including Central and South America. In the pregnant population studied, we found a prevalence of Chagas disease of 0.32 cases/1,000 persons screened over an 8.5-year period. In comparison, the risk of invasive group B *Streptococcus* (GBS) disease in the current era of universal maternal screening and treatment is similar, 0.3 case/1,000 live births. Although only 5% of maternal Chagas cases are estimated to be associated with perinatal transmission, the long-term illnesses and deaths associated with unrecognized Chagas disease are notable. This situation is similar to that of invasive GBS, which has a 3% case-fatality rate and a 5%–10% rate of sepsis meningitis, which will cause long-term neurologic effects in half of affected patients (*7*).

The incidence of Chagas disease varied over time. During the last year of the study period, an ≈3-fold increase in incidence occurred, although this finding is based on small numbers. Screening test methods remained constant throughout this period. We are unsure of the cause of this increase in incidence. Although it may have been secondary to continued immigration from Chagas disease–endemic areas, the small numbers of cases makes identification of factors difficult. 

In addition, the difference in ethnicity between the cohort with Chagas disease and the overall screened donors was significant, with an increase in self-identified Hispanic patients. These changes are consistent with the worldwide prevalence of the disease (*8*)

A strength of this study is its large sample size, particularly because the incidence of this disease is low. The study does, however, have several limitations. First, Chagas disease screening was limited to maternal donors of cord blood units donated to a public cord blood bank. Therefore, ascertainment bias is a possibility, despite use of a bilingual staff and consent and donor materials available in Spanish. However, this phenomenon would likely cause disease prevalence in the overall population to be underestimated. In addition, this sample primarily consists of donations across the state of North Carolina, with smaller proportions coming from Atlanta, Georgia; and Boston, Massachusetts. Therefore, these results may not be generalizable to all non–disease-endemic areas.

Our future work will focus on expanding the number of patients assessed by including other cord blood screening programs across the United States. We aim to determine patient demographics that will enable creation of targeted antenatal screening programs to reduce perinatal illness and death associated with congenital Chagas disease.
